# A Trans-Species Missense SNP in *Amhr2* Is Associated with Sex Determination in the Tiger Pufferfish, *Takifugu rubripes* (Fugu)

**DOI:** 10.1371/journal.pgen.1002798

**Published:** 2012-07-12

**Authors:** Takashi Kamiya, Wataru Kai, Satoshi Tasumi, Ayumi Oka, Takayoshi Matsunaga, Naoki Mizuno, Masashi Fujita, Hiroaki Suetake, Shigenori Suzuki, Sho Hosoya, Sumanty Tohari, Sydney Brenner, Toshiaki Miyadai, Byrappa Venkatesh, Yuzuru Suzuki, Kiyoshi Kikuchi

**Affiliations:** 1 Fisheries Laboratory, University of Tokyo, Hamamatsu, Shizuoka, Japan; 2 Department of Marine Bioscience, Fukui Prefectural University, Obama, Fukui, Japan; 3 National Research Institute of Aquaculture, Fisheries Research Agency, Minamiizu, Shizuoka, Japan; 4 Institute of Molecular and Cell Biology, A*STAR, Biopolis, Singapore, Singapore; Fred Hutchinson Cancer Research Center, United States of America

## Abstract

Heterogametic sex chromosomes have evolved independently in various lineages of vertebrates. Such sex chromosome pairs often contain nonrecombining regions, with one of the chromosomes harboring a master sex-determining (SD) gene. It is hypothesized that these sex chromosomes evolved from a pair of autosomes that diverged after acquiring the SD gene. By linkage and association mapping of the SD locus in fugu (*Takifugu rubripes*), we show that a SNP (C/G) in the *anti-Müllerian hormone receptor type II* (*Amhr2*) gene is the only polymorphism associated with phenotypic sex. This SNP changes an amino acid (His/Asp384) in the kinase domain. While females are homozygous (His/His384), males are heterozygous. Sex in fugu is most likely determined by a combination of the two alleles of *Amhr2*. Consistent with this model, the medaka *hotei* mutant carrying a substitution in the kinase domain of Amhr2 causes a female phenotype. The association of the *Amhr2* SNP with phenotypic sex is conserved in two other species of *Takifugu* but not in *Tetraodon*. The fugu SD locus shows no sign of recombination suppression between X and Y chromosomes. Thus, fugu sex chromosomes represent an unusual example of proto–sex chromosomes. Such undifferentiated X-Y chromosomes may be more common in vertebrates than previously thought.

## Author Summary

Diverse systems of sex determination have evolved independently in the animal and plant kingdoms. In vertebrates, so far four master sex-determining (SD) genes, *Sry*, *Dmrt1*, *Dmy*, and *Dm-W*, have been identified. These genes code for transcription factors and are located on only one of the sex chromosomes surrounded by nonrecombining regions. It is hypothesized that these sex chromosomes evolved from a pair of homologous chromosomes that diverged after acquiring the SD gene. We investigated the SD locus in fugu by high-resolution genetic mapping and association mapping. We found that a SNP that changes an amino acid (His/Asp384) in the kinase domain of anti-Müllerian hormone receptor type II (Amhr2) is perfectly associated with phenotypic sex. A combination of the two alleles of the SNP (homozygous females and heterozygous males) is likely to be responsible for sex determination in fugu. While these alleles are conserved in two other species of *Takifugu*, they are absent in the freshwater pufferfish, *Tetraodon*. Furthermore, Fugu *Amhr2* lies in a region that shows no evidence for recombination suppression between X and Y chromosomes. Thus, fugu sex chromosomes represent an unusual example of a pre-differentiated phase of sex chromosomes in vertebrates.

## Introduction

Diverse systems of sex determination have evolved independently in the animal and plant kingdoms [1]. The most prominent of them involves heterogametic sex chromosome systems where whole or a part of sex chromosomes are heterozygous in one sex (XY or ZW) and homozygous in the other (XX or ZZ). In many species, these sex chromosomes show distinctive morphology. It has been hypothesized that these sex chromosomes originated from a pair of autosomes that eventually diverged due to suppression of recombination [2], [3]. In addition, a master sex-determining (SD) gene often resides on one of the sex chromosomes. However, the SD gene varies between organisms, which underscores the independent evolution of SD and sex chromosome systems [1]. In vertebrates, previously four master SD genes were known; *Sry* [4] in therian mammals; *Dmrt1* in chicken [5]; *Dmy*, a duplicated copy of *Dmrt1* in medaka [6]; and *Dm-W*, a truncated copy of *Dmrt1* in *Xenopus laevis* [7]. All these SD genes code for transcription factors, belonging to either the Sox family (*Sry*) or the DM-domain family. When this paper was under review, two papers that identified non-transcription factors as vertebrate SD genes were published. One of these genes is a male-specific duplicated copy of the *anti-Müllerian hormone* (*amhy*) gene in the Patagonian pejerrey (*Odontesthes hatcheri*) [8]. The other is an allele of the *gonadal soma derived growth factor* (*Gsdf*) called *Gsdf^Y^*, located on the Y chromosome of *Oryzias luzonensis*, a species closely related to medaka [9]. Although studies of SD gene and sex chromosomes in vertebrates have provided significant insights into the evolution of sex-determining systems, our understanding is still very limited, given that sex chromosomes are known to exist in various states of differentiation [1] and that the SD genes remains to be identified in the vast majority of vertebrates.

In the present study, we investigated the SD region of the tiger pufferfish, *Takifugu rubripes* (fugu). Fugu is a large marine teleost and has an XX-XY sex determining system [10]. The availability of the whole genome sequence and a dense genetic map of fugu, combined with its compact genome size (400 Mb), allowed us to use the power of genetics to search for the SD gene in this wild species [11], [12].

## Results/Discussion

### Genetic Mapping

**Figure 1:**
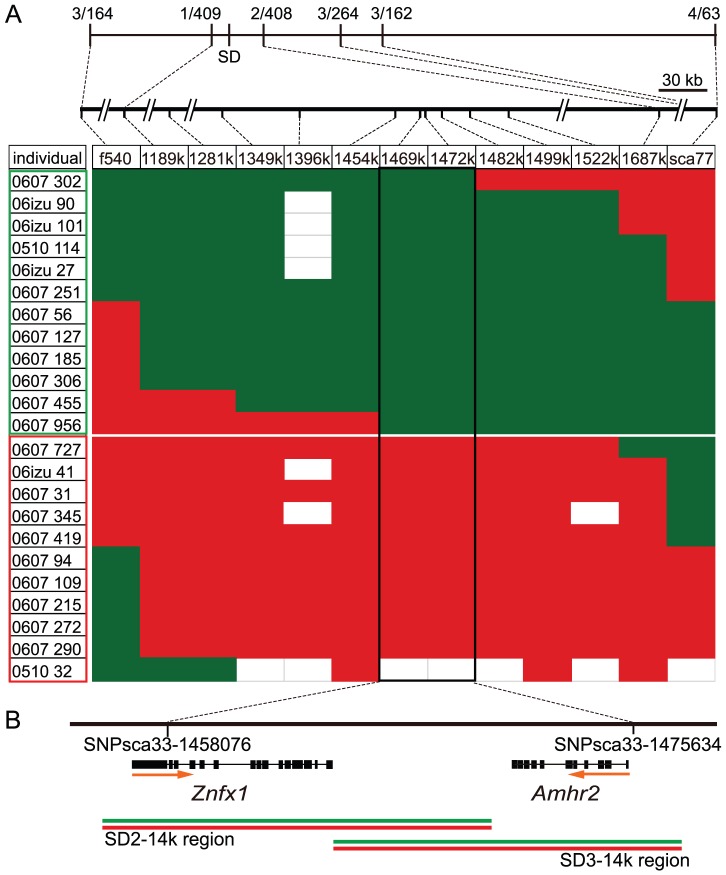
Fine mapping of the sex-determining locus in the pedigreed families of fugu. (A) Genetic map of the sex-determining (SD) region. The frequency of recombinant markers with the sexual phenotype observed in the initial genetic mapping is shown at the top of the linkage map. In the box below, each row is a recombinant individual found in the subsequent screening of 1034 additional siblings and those found in the initial mapping. IDs of males (green boxed) and females (red boxed) are shown in the first column, followed by allele type at markers along the sex chromosome. Alleles shown in red and green are transmitted to nonrecombinant females and males, respectively. Empty blocks indicate that genotypes are non-informative or not assigned. Marker names are indicated at the top of column and their genomic positions are shown above. A combination of markers sca77 and f540 or sca77 and 1189 k is used for screening recombinants from 1034 siblings. The boxed 17.5 kb region shows perfect correlation between the haplotype and phenotypic sex. (B) Schematic diagram of the SD region and gene structures. The positions of SNPs defining the 17.5 kb SD region and two predicted genes in this region are shown. Orange arrows indicate the transcriptional orientation of the genes. Green and red lines indicate the position of genomic DNA clones that were amplified by PCR, cloned into plasmids and sequenced. Two clones each for the genomic regions SD2–14k and SD3–14k were sequenced from X (red line) and Y (green line) chromosomes.

Our previous genome-wide linkage mapping in fugu has shown that the fugu SD region is restricted to a small segment of chromosome 19 flanked by large autosome-like regions [10], [12], [13]. In the fugu genome assembly (version 4), this locus includes four scaffolds of total length 5387 kb, containing ∼300 potential protein-coding genes [10]. Since a contiguous physical map of this region was not available, we first increased the resolution of the genetic map to determine the precise positions of the four scaffolds (Table S1). We next produced large numbers of fugu siblings and performed extensive linkage analyses to reduce the genomic interval of the SD region (Figure 1A, Table S2). We first narrowed down the SD region by analyzing 411 individuals and then searched for recombinants in the SD region in 1034 additional siblings. Comparison of the genotype of markers in the SD region and the sex of the 23 identified recombinants localized the SD region to a short stretch of 17.5 kb in which two protein-coding genes, *NFX1-type zinc finger-containing 1* (*Znfx1*) and *anti-Müllerian hormone receptor type II* (*Amhr2*), are predicted (Figure 1B). Znfx1 is a transcription factor of unknown function that is expressed in a wide range of tissues in mammals including central nervous system, kidney, lung, muscle, ovary, testis, pancreas and thyroid [14], whereas Amhr2 is a receptor in the anti-Müllerian hormone (Amh) pathway that plays an important role in development and maintenance of reproductive organs in mammals and other vertebrates [15].

Since previously known SD genes in vertebrates are specific to one of the sex chromosomes (Y, Z or W) that shows varying degrees of sequence differentiation with the other sex chromosome [4]–[7], we examined whether the fugu SD region contains a distinct male-specific segment by PCR amplification, cloning and sequencing of the entire 17.5 kb SD region of two males chosen from experimental families (Figure 1B). We then determined whether the genomic clones were derived from X or Y by using the markers polymorphic in the families. The sequences of the X and Y derived clones covering the 17.5 kb SD region were more than 99% identical (99.4% for SD2–14k region and 99.6% for SD3–14K region, Figure S1) indicating that the male sex in fugu is determined by a small difference in the genomic sequence.

### Association Mapping

**Figure 2:**
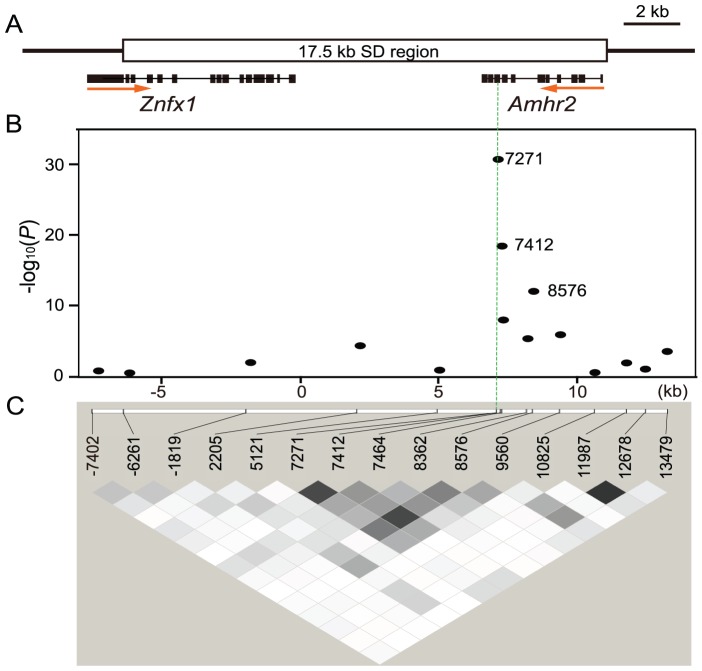
Association mapping of the sex-determining locus in a natural population of fugu. (A) Schematic diagram of the SD region. The 17.5 kb SD region and both of the only two predicted genes are shown. (B) Plot of −log_10_ (*P* value) versus chromosome position for association test of the SD region. Position starts from the 5′ end of the genomic clone covering the entire *Amhr2* gene shown in Figure 1B (SD3–14k region). SNP 7271 in *Amhr2* showed perfect correlation with phenotypic sex (green dotted vertical line). Bonferroni correction gives a significance threshold of −log_10_(*P*) = 3.2. (C) Linkage disequilibrium (LD) around the SD region. The square of the correlation coefficient (r^2^) is estimated for each pairwise comparison of SNPs, with darker grey indicating stronger LD (white, r^2^ = 0; shades of grey, 0<r^2^<1; black, r^2^ = 1).

**Figure 3:**
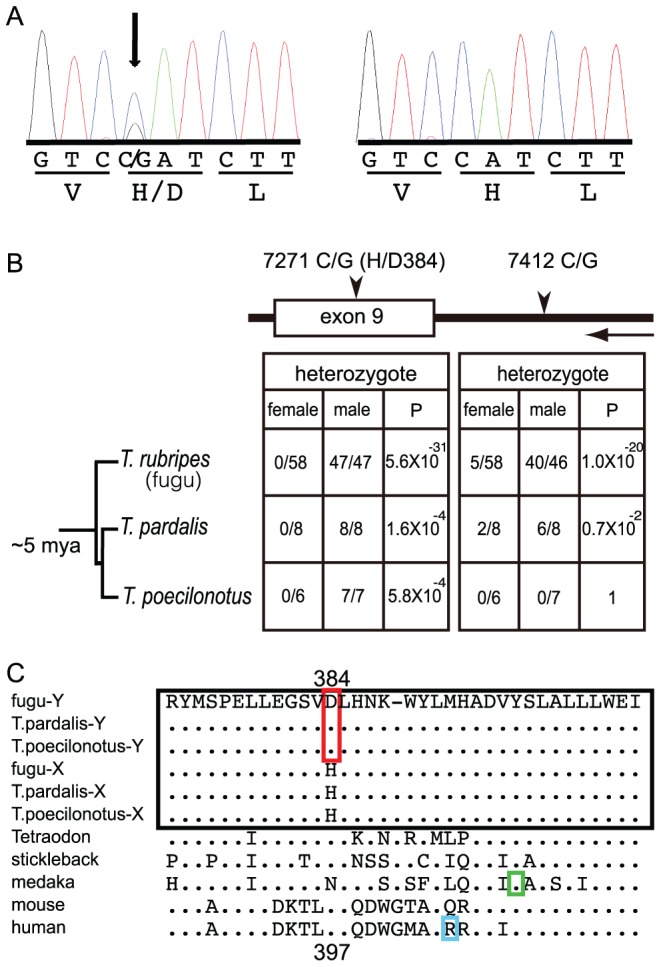
A trans-specific SNP in *Amhr2* is correlated with phenotypic sex in *Takifugu*. (A) Sequence traces of *Amhr2* from a male (left) and a female (right) fugu. The male is heterozygous at the non-synonymous SNP site that converts His384 codon into Asp384 codon. (B) Sexual phenotypes, genotypes and statistical significance of trans-species SNPs. Proportion of heterozygotes among females and males in three species of *Takifugu* are shown. *P*-values are from 2×2 Fisher's exact test of the genotype count data. A horizontal arrow indicates the transcriptional orientation of the genes. (C) Comparison of a part of kinase domain sequences of Amhr2 from various vertebrates. The sequences from *Takifugu* species are boxed in black. The amino acid specific to the male chromosome of *Takifugu* is boxed in red. His/Asp384 of fugu Amhr2 corresponds to Asp397 of human AMHR2. The position of the amino acid mutation in the medaka (*hotei*) that results in a female phenotype when homozygous is boxed in green. The amino acid position of a natural mutation leading to the loss-of-function of human AMHR2 is boxed in cyan.

To precisely pinpoint the SD gene, we employed association mapping utilizing ancestral recombination in a wild population of fugu (Figure 2). SNPs and other polymorphisms were screened by sequencing the entire SD region of seven males from seven experimental crosses, the 5′region of SD3 for X and Y in one male from an eighth family, and the 5′ region of SD3 for only X in a male from the ninth family (Figure S1, Table S3). In this analysis, we noted that only one of the variants, SNP 7271 (C>G), residing within exon 9 of *Amhr2* is heterozygous in all males (Figure 3A, Table S3A and S3B). This SNP is located within the kinase domain of Amhr2 and changes an amino acid from His384 to Asp384 (Figure 3). Additional genotyping of this SNP in nine families showed perfect correlation with the male phenotype (Table S2). While 100% of males (41/41) were heterozygous (C and G) at this position, all females (44/44) were homozygous for the C allele.

Apart from this SNP, we did not find any other SNP or other types of variants (repeats, indels, etc.) that was heterozygous in any male (Table S3). We further investigated this SNP and other SNPs chosen from the 17.5 kb SD region and its neighboring regions (∼2 kb upstream harboring three SNPs and ∼600 bp downstream containing one SNP) in a natural population consisting of 58 females and 47 males (Figure 2). We first calculated linkage disequilibrium (LD) between all SNPs examined and found that the overall extent of LD in the SD region is very low (Figure 2C). This pattern suggested that recombination has not stopped in the region containing the fugu SD gene, unlike the previously characterized SD regions in other vertebrates [3]. Indeed, there is no significant LD even between markers that are 1 kb apart, thus providing a remarkably high resolution for the SD region. We then examined the association between each SNP and the sex phenotype. Consistent with the results of the small-scale analysis, SNP 7271 showed perfect association with the male phenotype (*P* = 5.6×10^−31^) (Figure 2B). In addition, its neighboring variant, SNP 7412 residing in intron 8 also showed a strong association with the male phenotype (*P* = 9.9×10^−19^). However, the latter is unlikely to be responsible for SD because 9 out of 105 wild fish showed discordance in their genotypes and phenotypic sex. The distribution of its genotype was 53 GG and 5 CG in females, and 4 GG, 40 CG, 2 CC and 1 undetermined in males. The association of SNP 7412 to phenotypic sex can be explained as an indirect association due to its close proximity to SNP 7271 (141 bp apart, r^2^ = 0.7) that shows a perfect correlation with the phenotypic sex (Figure 2 and Figure S2). The results of the association mapping studies together with those of variant screening suggest that the SNP 7271 (C>G) is the sole polymorphism in the 17.5 kb SD region that shows perfect correlation with phenotypic sex.

### Trans-Species Comparison

Conservation of SNPs between species is rare, unless they are under some selective constraint, or that the species are so closely related that they still share their ancestor's variants. Thus, trans-species analysis offers an additional test to verify the correlation of SNP7271 to phenotypic sex in fugu [16], [17]. To this end, we investigated two other wild species of *Takifugu*, *T. pardalis* (n = 8 for each sex) and *T. poecilonotus* (n = 6 females and 7 males), that diverged from *Takifugu rubripes* approximately 5 million years ago (mya) [18]. We PCR amplified and sequenced a part of the *Amhr2* gene (intron 8, exon 9 and intron 9) from them. Comparison of the sequences revealed that while SNP 7271 is present in both species, the intronic SNP 7412 is present only in *T. pardalis* (Figure 3B). More importantly, examination of all SNPs around exon 9 of *Amhr2* from the two species indicated that SNP 7271 is the sole conserved polymorphism that shows perfect correlation with the sexual phenotype (Figure 3B and 3C and Figure S3, Table S4). These results strongly suggest that SNP 7271 is a trans-species SNP and is likely to be a causative variant for sex determination in the three species of *Takifugu*. Interestingly, this SNP is absent in the green spotted freshwater pufferfish, *Tetraodon nigroviridis* (n = 4 for each sex) which diverged from fugu approximately 40 to 70 mya [18],[19]. This indicates that the sex-determining polymorphism, SNP 7271 in *Amhr2*, evolved in a common ancestor of the three *Takifugu* species after it diverged from the *Tetraodon* lineage.

### Structure and Identity of Fugu *Amhr2* Gene

To confirm the exon-intron organization of the *Amhr2* gene predicted in the fugu genome assembly, we amplified full-length cDNA of fugu *Amhr2* ^H384^ and *Amhr2* ^D384^ using cDNA from the testis as a template (accession number AB618627 in DDBJ). Sequencing of these products indicated that fugu *Amhr2* comprises 11 exons, and encodes a protein of 514 amino acids (Figure S4A). The fugu protein is 28% identical to the human AMHR2 with 41% identity in the kinase domain (Figure S4A). The fugu *Amhr2* gene is embedded in a syntenic block of genes that is conserved in *Tetraodon*, stickleback as well as in human (Figure S4B). This suggests that the fugu gene is an ortholog of *Amhr2* in these species and that it was not transposed recently into the fugu SD locus in the *Takifugu* lineage. To further confirm its identity, we carried out phylogenetic analysis of Amhr2 and its related proteins from various vertebrates. This analysis confirmed that the fugu gene is an ortholog of *Amhr2* in other vertebrates (Figure S4C). Searches of the draft genome assemblies of fugu, *Tetraodon*, stickleback and medaka identified only a single copy of *Amhr2*, suggesting that following the fish-specific whole genome duplication event in the teleost ancestor [20], [21], the duplicated copy of *Amhr2* may have been lost in these teleosts.

### Expression Pattern of Fugu *Amhr2*

**Figure 4:**
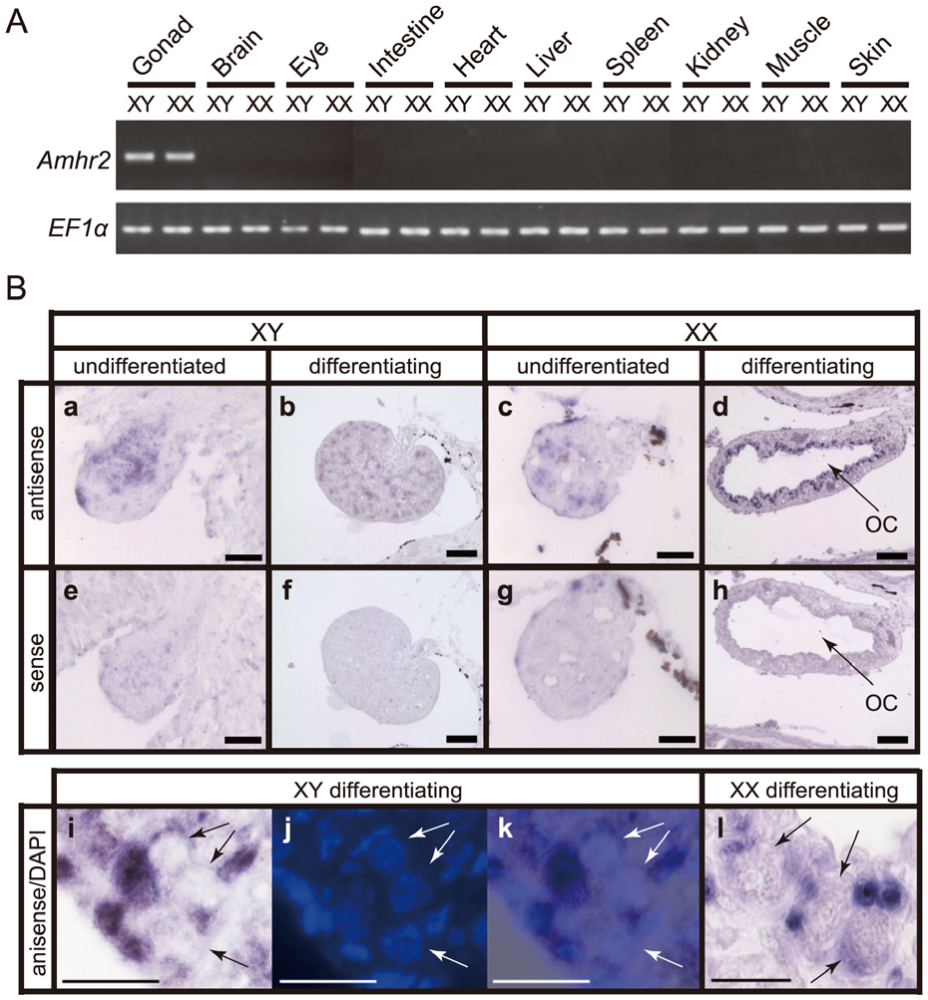
Expression pattern of *Amhr2* in fugu. (A) RT-PCR analysis of the tissue distribution of Amhr2 mRNA in juvenile fugu at 147 days after fertilization (dpf). EF1a was used as an internal control. PCR analysis was performed for three males and females, and only representative results for a male and a female are shown. (B) *In situ* hybridization on undifferentiated and differentiating gonads. Genotypic sexes are shown as XY and XX. *In situ* hybridization with antisense probe (a–d, and i–l) and sense probe (e–h) for *Amhr2* are shown. (a, e) Undifferentiated gonad in XY fish at 62 dpf. (b, f) Differentiating gonad in XY fish at 90 dpf. (c, g) Undifferentiated gonad in XX fish at 62 dpf. (d, h, l) Differentiating gonad in XX fish at 90 dpf. The ovarian cavity (OC) is apparent only in differentiating XX gonad. The large and round germ cells (arrows in l) were evident under DIC optics. (i–k) Differentiating gonad in XY fish at 126 dpf. The germ cells containing large and round nucleus (arrows) were apparent in DAPI stained (j) and overlay (k) images. Scale bar, 20 µm (a, e, c, g), 50 µm (b, f, d, h) and 10 µm (i–l).

In mammals, Amhr2 is responsible for the regression of the Müllerian duct in males [15]. Loss-of-function of this gene in male mouse leads to a partial hermaphrodite having a uterus and an oviduct together with the testis [15]. However, in medaka, a homozygous mutation in exon 9 of *Amhr2* (Tyr390Cys) (*hotei* mutant) results in complete sex reversal in half of the genetic males [22]. These results indicate that although *Amhr2* is not essential for formation of the testis in mammals, in fishes *Amhr2* has the potential to influence the decision of the bipotential gonad to become either an ovary or a testis. If the *Amhr2* gene indeed resides at the top of the sex determination pathway in fugu, it should be expressed before the differentiation of ovary and testis. However, it need not be expressed in a sex-specific manner like the mammalian *Sry* or medaka *Dmy*, because the sex-specific signaling can be produced by the male-specific isoform, Amhr2^D384^. Nevertheless, to verify if there is differential expression of *Amhr2* between males and females, we analyzed *Amhr2* expression in fugu by RT-PCR and in situ hybridization. Gonadal sex differentiation in fugu begins at 8–9 weeks after fertilization when juveniles attain a body length of approximately 25 mm [23]. The first sign of the morphological differentiation of ovary and testis is the formation of the ovarian cavity [23], [24]. The RT-PCR analysis revealed that *Amhr2* is expressed in the differentiating ovary as well as the testis of juvenile fugu (Figure 4A; n = 3 for each sex). The *in situ* hybridization indicated that *Amhr2* is expressed in both sexes before the morphological differentiation of gonads (Figure 4B; n = 3 for female and n = 4 for male), and subsequently in somatic cells surrounding the germ cells of the differentiating gonads (Figure 4B; n = 5 for each sex at 90 dpf and n = 2 for each sex at 126 dpf). A similar expression pattern of *Amhr2* is also reported in medaka [25]. These results provide further support for a role for *Amhr2* in the sex determination of fugu.

### Effect of Missense SNP in Kinase Domain of Amhr2

Amhr2 is a type II receptor for the TGF-ß family of proteins and contains a single transmembrane domain and a serine/threonine kinase domain [26]. Upon binding to Amh, Amhr2 recruits and phosphorylates a type I receptor(s) that then transduces signals by phosphorylating Smad proteins which in turn regulate transcription of downstream genes in mammals [26]. SNP 7271 is located within the kinase domain of Amhr2 (Figure 3C) that is responsible for the phosphorylation. Five natural mutations in the kinase domain of Amhr2 in human [26] and one induced mutation in medaka (Tyr390Cys) [22] (see Figure S4D) resulted in a loss-of-function phenotype of Amh/Amhr2 signaling. Thus, the kinase domain is critical for the function of Amhr2. Since Asp384 is conserved in *Tetraodon*, stickleback, mouse and human (in medaka, Asp is replaced with Asn; Figure 3C), we propose that *Amhr2* ^H384^ is a derived allele in *Takifugu* that causes a female phenotype when homozygous.

**Figure 5:**
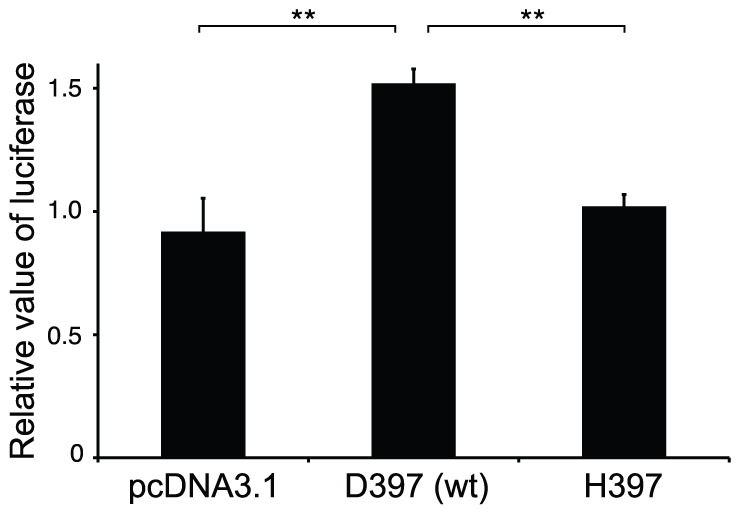
Mediation of AMH signaling by wild-type and mutated human AMHR2 (His397) constructs. Each assay was done in triplicate, and the results were expressed as mean ± SEM. Human AMHR2^H397^ mediated significantly less signaling compared to wild-type human AMHR2 following the addition of human rAMH. Double asterisks indicate significant difference (*P*<0.01) between groups.

To determine the effect of Asp to His substitution in the kinase domain of *Amhr2* on the activation of Smad proteins in fugu, we first tried an antibody that can recognize the activated Smad 1/5/8 in mammals and zebrafish on sections of the differentiating testis of fugu by immunohistochemistry [27]. However, the antibody was not effective in fugu. We then used a mouse teratocarcinoma cell line, P19, which contains all the molecules including type I receptor required for Amh signaling except Amhr2 [26] to assay the effect of substitution of fugu Amhr2 on the activation of Smad1. However, both fugu *Amhr2* ^D384^ and *Amhr2* ^H384^ failed to elicit Smad1 activity in this cell line (presumably due to the incompatibility with the mouse type I receptors) (data not shown). Because there are no comparable cell lines from fugu or other fishes, we introduced the His384 mutation into human *AMHR2* (His397) and tested the human construct in P19 cells. Interestingly, the human mutant hAMHR2^H397^ mediated significantly less signaling (Smad1 activation) compared to hAMHR2^D397^ (Figure 5). This result suggests that fugu *Amhr2* ^H384^ is an allele with reduced function compared to *Amhr2* ^D384^ and the homozygous form of this less-effective allele facilitates the formation of ovary, whereas the heterozygous form of the alleles, *Amhr2* ^H384^ and *Amhr2* ^D384^, promotes development of testis. This implies that *Amhr2* ^D384^ is a dominant allele. This interpretation is consistent with the male to female sex-reversal in the medaka *Amhr2* homozygous mutant (*hotei*) that has a loss-of-function mutation in the kinase domain (Tyr390Cys) [22], located 13 positions downstream of fugu Asp384 (see Figure 3C).

One possible mechanism of sex determination by Amh/Amhr2 signaling is that it decreases the number of germ cells, which in turn promotes the development of testis. This hypothesis is based on the observation that in the medaka *hotei* mutant over-proliferated germ cells were associated with ovary formation in males [22]. On the other hand, inhibition of primordial germ cell migration into gonads by knockdown of the chemoattractant receptor gene, *cxcr4*, resulted in the formation of testis-like structure in females [28]. Consistent with this finding, high temperature-induced germ cell degeneration in fugu is associated with masculinization of ovarian somatic cells [29]. Another possibility is that the Amh/Amhr2 signaling could antagonistically act on the aromatase activity as reported in mammals [30]. Enhanced estrogen synthesis by aromatase is known to function as a natural inducer of ovarian differentiation in fishes [31]. Thus suppression of aromatase activity by Amh/Amhr2 signaling could lead to the formation of testis in fugu.

### Allelic Sex Determination by a Non-Transcription Factor

In vertebrates, previously four master SD genes were known: *Sry* in therian mammals [4]; *Dmrt1* in chicken [5]; *Dmy* in medaka [6]; and *Dm-W* in *Xenopus laevis* [7]. They all code for transcription factors and are able to regulate the expression of other genes in the sex-determination pathway. By contrast, the *Amhr2* gene associated with the sex determination in fugu codes for a growth factor receptor. This suggests that any gene that lies in the sex-determination pathway has the potential to be recruited for triggering sex determination. Furthermore, unlike the known vertebrate SD genes that reside on only one of the pair of sex chromosomes, fugu *Amhr2* is located on both of the homologous chromosomes. This suggests that phenotypic sex in fugu is determined by a combination of the two allelic variants of *Amhr2* gene.

When this paper was under review, two other novel candidates for vertebrate SD genes were reported, and both are in fishes. The first one is a male-specific, duplicated copy of the *Amh* gene (*amhy*) implicated in testicular development of the Patagonian pejerrey [8]. Morpholino-mediated knock down of *amhy* (and presumably *amh*) resulted in male to female sex reversal in 22% of fishes carrying the duplicated copy of *Amh*. Interestingly, *Amh* codes for a hormone that is a ligand for Amhr2. The other SD gene, *Gsdf^Y^* identified in *O. luzonensis* [9], is an allele similar to the sex-determining *Amrh2* allele in fugu. The promoter analysis of this gene showed that the allelic difference involves 6 to 9 nucleotide difference in the binding site for a steroidogenic factor 1 (SF1) and results in male-specific high expression of the *Gsdf^Y^* allele. By contrast, the polymorphism in fugu is in the coding sequence and affects the function of the protein. Nevertheless, it is interesting that all these three novel fish sex-determining genes are components of the TGF-ß signaling pathway. These findings underscore the critical role of TGF-ß signaling, a hitherto unappreciated pathway, in gonadal sex determination in vertebrates. It is possible that additional members of this pathway may be involved in sex determination in other teleosts and other non-mammalian vertebrates that have experienced a recent turnover of sex chromosomes.

### Establishment of a Recessive Allele as a New SD Gene

Several models have been proposed to explain the evolution of new sex-determination mechanisms. These include random genetic drift [32], pleiotropic selection favoring new SD alleles [33] and sexually antagonistic selection [34]. The last model begins with an autosomal locus segregating alleles that have different relative fitness in males and females. Selection will favor an increase in the frequency of any new SD locus that is linked to these sexually antagonistic alleles. When the new SD allele is recessive, like the fugu *Amhr2* ^H384^, it cannot increase in frequency quickly. However, a recessive allele could first increase in frequency by drift. Once its frequency reaches a certain level, sexually antagonistic selection at the sex-linked locus can lead to the recessive feminizing allele to spread to fixation, resulting in the disappearance of the previous female determiner such as a dominant allele on the W chromosome. This could lead to heterogamety switch from ZW/ZZ to XX/XY. Since Amh/Amhr2 signaling plays a role downstream of the SD gene in many vertebrates including mammals, birds and medaka [15], [22], [35], the fixation of such a downstream factor as the new SD locus might explain the later onset of the gonadal dimorphism, which occurs around 8–9 weeks after fertilization in fugu.

### Proto–Sex Chromosomes

The theory of sex chromosome predicts that when an existing SD gene is replaced by a new SD gene, the young sex chromosomes lack a nonrecombining region. Such a region subsequently evolves through suppression of recombination leading to the divergence of the sequences of the homologous sex chromosomes [3]. The suppression of recombination is generally mediated by accumulation of repetitive sequences and/or inversion of chromosomal segments spanning the newly arisen SD gene/locus [36]. The sex chromosome theory thus implies that just after a new SD gene has replaced an existing one, the two homologous chromosomes show little divergence in their sequences even around the SD gene. However, no such instance of highly similar sex chromosome pair is known in vertebrates except in fugu, and the recently reported *O. luzonensis* [9]. The two youngest known SD genes in vertebrates, medaka *Dmy* and *Xenopus Dm-W*, arose by segmental duplications and are embedded within recognizable nonrecombining regions. In medaka, the Y-specific nonrecombining region, originally derived from the duplication of a 43-kb fragment of the *Dmrt1* locus, has grown to 258 kb by accumulating 137 kb of repetitive sequences [37]. The W-specific region of *Xenopus*, likely derived from a duplication of the *Dmrt1* locus, is flanked by 3 to 4 kb of nonrecombining regions [7], [38].

In fugu, although the recombination ratio in males is reduced compared to females even in regions around SD locus (Table S1), our genetic analysis did not identify any nonrecombining region in the SD locus (Figure 1 and Figure 2). This is consistent with our previous observation that the suppression of recombination in males relative to females is no greater around the sex-determination locus than on autosomes [12]. Furthermore, sequence comparison between the two SD chromosomes did not reveal any inversions or large scale accumulation of transposons and other repetitive elements (Figure 1 and Figure S1). Overall, the fugu SD region contains very little repetitive sequences (1.8% of retroelements and 2.3% simple repeats). Therefore, the SD chromosomes of fugu provide an unusual example of proto-sex chromosomes. The SD locus in therian mammals, medaka and *Xenopus laevis* are associated with duplication events, which can explain recombination suppression. However, in the case of Takifugu species, since sex is determined by just a single nucleotide difference, the SD locus might have eluded recombination suppression. Although there is little information regarding recombination in the SD locus of *O. luzonensis* [9], it would be interesting to determine if recombination around this SD gene still occurs, as in fugu.

Stock et al. [39] have recently shown that three species of European tree frogs that diverged 5 to 7 mya share the same pair of sex chromosomes with complete absence of X-Y recombination in males. Yet, the sequences of sex-linked loci are very similar between the X and Y chromosomes. Since phylogenetic analysis showed that the X and Y alleles clustered according to species rather than gametologs, the authors proposed that the similarity of the sequences at the sex-linked loci is maintained by occasional X-Y recombination, presumably occurring in sex-reversed XY females. Although this model may explain the sequence similarity between X and Y at the SD locus in fugu, the frequency of XY fugu females appears to be very low. We have not encountered any XY female in our genetic experiments. Moreover, attempts at producing XY females by hormonal or temperature manipulation have proven to be unsuccessful [29], [40]. Thus, the extent of the contribution of sex-reversed XY females in maintaining similarity at the SD locus of fugu is unclear.

### Conclusion

We investigated the SD locus in fugu by high-resolution genetic mapping and association mapping. We found that a missense SNP in the kinase domain of Amhr2 that changes an amino acid is the sole polymorphism perfectly correlated with phenotypic sex. Our results suggest that a combination of the two alleles of *Amhr2* is responsible for sex determination in fugu. The pattern of LD across the fugu SD locus indicates the absence of a nonrecombining region. Thus, the sex chromosomes of fugu represent a unique example of proto-sex chromosomes in vertebrates. Since genetic mapping studies of sex-determination loci in diverse taxa of vertebrates have indicated that vertebrates such as fishes, reptiles and amphibians have experienced a rapid turnover of sex chromosomes [1], the pre-differentiated sex chromosomes found in fugu may be more common among these vertebrates than previously thought.

The successful identification of the candidate sex-determining SNP in this study relied essentially on a sub-gene-level resolution of the association mapping owing to the low degree of LD in a wild population like fugu. In human populations, this method usually provides resolution at the level of only one or a few genes (tens to hundreds of kb) [41]. The resolution is even lower in laboratory mouse and domestic animals due to the strong LD across the genomes caused by a small effective population size [42]. By contrast, a wild species with a large effective population size is likely to show a low degree of LD across its genome. Thus the use of association mapping should greatly facilitate the identification of SD genes and chromosomes in the wild populations of non-model vertebrates.

## Materials and Methods

### Pedigreed Families for Linkage Mapping

To produce siblings, we crossed a fugu male with a fugu female as described previously [13]. Details of the families used in linkage mapping are shown in Table S2. Sex was determined by histological examination of the gonads for fishes aged 2–10 months and by visual inspection for older fish. The microsatellite loci were chosen by scanning scaffolds as described previously [13]. Primer sequences for microsatellite markers are listed in Table S5. Genotyping with microsatellite markers was performed as described previously [13]. This project was conducted in accordance with the Regulation for Animal Experiments of the University of Tokyo.

### DNA Sequences of the SD Region from X and Y Chromosomes

We generated nine families and used males from these pedigrees for generating complete sequences of the SD region from X and Y chromosomes (Figure S1, Table S2). Genomic clones covering *Znfx1* (genomic region of SD2–14k) and *Amhr2* (genomic region of SD3–14k) were obtained from the XY single male by PCR using KOD FX (Toyobo) reagents suitable for amplifying large genomic fragments (Figure 1B and Figure S1). Approximately 50 ng of genomic DNA was used as a template in 25 µl PCR reaction. PCR amplification was performed using KOD DNA polymerase (Toyobo) with the primer pair SD2–14.7kF and SD2–14.7kR for SD2–14k genomic region, and the primer pair 33–1464k340F and 33–1464k13469R for SD3–14k genomic region. The cycling conditions were 36 cycles of 94°C for 10 s and 70°C for 12 min. Primer sequences are given in Table S5. DNA fragments were cloned by using TOPO XL PCR cloning kit (Invitrogen) or In-Fusion Advantage PCR cloning Kit (Clontech). The clones from X and Y chromosome were distinguished by SNP7271 or satellite marker 1469 K and were subjected to sequencing. To identify candidate polymorphic sites and obtain variants for linkage disequilibrium analysis, we cloned genomic DNA covering *Amhr2* gene (the 5′ and 3′ regions of SD3, Figure S1) from males from seven independent families. In addition, we cloned genomic DNA covering the 5′ region of SD3 from two males from two other independent families. We sequenced theses clones and identified variants among them (Table S3). To avoid cloning artifacts, we followed the method described by Saitoh and Chen [43]. For variants found in only one clone, two additional clones were sequenced to obtain the consensus sequence. For the region covering *Znfx1* (genomic region of SD2–14k), we directly sequenced the PCR products from ten males and determined DNA sequences for at least seven individuals from ten males.

### Association Test

We studied a natural population of fugu consisting of 58 females and 47 males from off shore areas around the mid-west part of Japan. Genotyping was done using TaqMan or restriction fragment length polymorphism (RFLP) analysis (Table S4). We calculated association for SNPs having a minor allele frequency (MAF)>0.1 and a call rate >99% with phenotypic sex in fugu using Haploview program 4.1 [44]. Uncorrected *P*-values and *P*-values with 1,000,000 permutations are reported. We also tested the association between genotypes and phenotypic sexes for a recessive model of penetrance in which homozygosity of one of the alleles is required for phenotypic female based on the previous studies that have suggested that the sex of fugu is determined by an XX-XY system [10]. Linkage disequilibrium plot of r^2^ and D′ was generated using Haploview.

### cDNA Cloning and Phylogenetic Analysis

cDNA of the fugu *Amhr2* ^H384^ and *Amhr2* ^D384^ were cloned from the testis using SMART RACE cDNA amplification Kit (Clontech) and sequenced completely. Multiple sequence alignment and NJ tree of fugu Amhr2 and its related proteins from various vertebrates were generated using ClustalW [45].

### Trans-Species Association Test

We obtained *T. pardalis* (n = 8 for each sex) and *T. poecilonotus* (6 females and 7 males) from Lake Hamana, Japan. To determine exon 9 sequence of *Amhr2* and its neighboring regions, we first amplified the genomic region using primer SD3exon8F and SD3exon10R, and sequenced the PCR product directly. *Tetraodon nigroviridis* specimens (n = 4 for each sex) were obtained from a supplier in Philippines, and the sequence of *Amhr2* exon 9 and its neighboring regions were determined after PCR amplification with primers Tet-SD3exon8F and Tet-SD3exon10R. See Table S5 for primer sequences.

### RT–PCR

One microgram of total RNA each from the gonad, brain, eye, intestine, heart, liver, spleen, kidney, trunk muscle and skin of fugu at 147 days post-fertilization (dpf) was used for synthesizing first-strand cDNA. PCR amplification was performed with the primer pair RT-SD3exon9F and RT-SD3exon9R, and the primer pair EF1α-F and EF1α-R. The cycling conditions were 40 cycles of 94°C for 10 s, 58°C for 5 s, and 72°C for 30 s. See Table S5 for primer sequences.

### *In Situ* Hybridization

Juveniles at 62, 90 and 126 dpf from a full-sib family were dissected along the ventral midline and fixed in Bouin's solution or 4% paraformaldehyde at 4°C overnight. Fixed samples were dehydrated in graded ethanol, embedded in paraffin and sectioned serially at 5 µm thickness. After rehydration, sections were subjected to hybridization. The probes were transcribed from a fugu *Amhr2* cDNA construct using DIG RNA labeling kit (Roche). Signals were detected by immunoreaction with alkaline phosphatase-conjugated anti-DIG antibody and NBT/BCIP solution (Roche). The genotypic sexes of all fish were determined by SNP7271 while phenotypic sex of fish at 90 and 126 dpf were determined by the formation of the ovarian cavity. The ovarian cavity was not seen in any fish at 64 dpf with the body length ranging from 19 to 22 mm (n = 4 for XY fish and n = 5 for XX fish).

### Constructs for Luciferase Assay

The coding region of human *AMHR2* was PCR amplified using a cDNA clone (MHS4426-99239518, Open Biosystems) as a template with hAMHRII-F and hAMHRII-R primers. The amplicon was ligated into the pcDNA3.1 vector (Invitrogen). Site-directed mutation was carried out using KOD-Plus-Mutagenesis Kit (Toyobo) with hAMHRII-D397H-F and hAMHRII-D397H-R primers. The coding region of mutated hAMHRII was amplified by PCR and ligated into the pcDNA3.1. Coding region of human Smad1 was amplified by PCR using Smad1f and Smad1r primers with pCMV5 Smad1 (Addgene) as a template. The amplicon and the pBIND vector (Promega) were ligated to generate the construct Gal4-Smad1/pBIND. The constructs were confirmed by sequencing. Construction of 5xGal4-tk-luc construct has been reported previously [46]. See Table S5 for primer sequences.

### Cell Culture of P19 and Luciferase Assay

Mouse embryonal carcinoma P19 cells were cultured in Minimum Essential Medium (αMEM, Sigma M8042) containing 2 mM L-Alanyl-L-Glutamine, 7.5% FCS and 2.5% FBS at 37°C under 5% CO_2_. P19 was seeded in 96-well plates at 1×10^4^ cells/100 µl medium 24 h prior to transfection. Transfection was conducted using Lipofectamine LTX (Invitrogen). 5xGal4-tk-luc, Gal4-Smad1/pBIND and empty pcDNA3.1, wild-type or mutated human AMHR2 in pcDNA3.1 were transfected. After addition of the recombinant human MIS (rhMIS, R&D Systems, 2 µg/ml), the cells were cultured for 24 h. Luciferase activity was determined using Dual Luciferase Kit (Promega). Firefly luciferase activity was normalized to *Renilla* luciferase activity. Each experiment was done in triplicates and the average of normalized activity was calculated. Three independent transfections were carried out, and values obtained by rhMIS treatment were divided by those obtained without rhMIS treatment. Data were expressed as mean ± SEM, with mean values comprising values from three independent assays: 3 per assay (9 per treatment). Luciferase activity was compared by One-way ANOVA (two-tailed, *P*<0.05). The Holm-Sidak test was applied to measure the differences among means for those cell types (overall significance level *P* = 0.05).

## Supporting Information

Figure S1 Screening for variants in the 17.5 kb SD region of fugu. We demarcated 4 genomic regions for sequencing: SD2–14K region, SD3–14k region, the 5′ region of SD3 and the 3′ region of SD3. Red and green bars indicate genomic clones of X and Y chromosomes, respectively, from XY males belonging to independent families. The derivation of clones was determined by the transmission patterns of polymorphic marker allele types. Sequences of the regions covered by blue and orange arrows were determined by direct sequencing of PCR products. Sequences were obtained from 7 to 10 XY males belonging to independent families for the blue region, whereas sequences from 4 to 10 XY males were determined for the orange region. The orange region is located outside of the 17.5 kb SD candidate region. The results of screening are shown in Table S3.
(EPS)

Figure S2 Linkage disequilibrium of the SD region of fugu. r^2^ and D′ values ×100 estimated for each pairwise comparison of SNPs are shown in plots (A) and (B), respectively. Darker grey in (A) indicates higher r^2^ (white, r^2^ = 0; shades of grey, 0<r^2^<1; black, r^2^ = 1), while darker red in B indicates higher D′ (white, D′<1 and LOD<2; blue, D′ = 1 and LOD<2; pink, D′<1 and LOD≥2; red, D′ = 1 and LOD≥2).
(TIF)

Figure S3 SNP 7271 is the sole conserved SNP perfectly correlated with phenotypic sex among *Takifugu* species. We screened variants in exon 9 of *Amhr2* and its flanking introns from 16 individuals (8 females and 8 males) of fugu, 16 individuals (8 females and 8 males) of *T. pardalis* and 13 individuals (7 females and 6 males) of *T. poecilonotus*. We combined all SNP data and calculated association of allele frequency with phenotypic sex using Haploview program [44]. Uncorrected *P*-values and *P*-values with 1,000,000 permutations are reported. Bonferroni correction of the 13 SNPs tested gives a significance threshold of −log_10_(P) = 3.2.
(EPS)

Figure S4 Trans-species comparison of Amhr2. (A) Comparison of Amhr2 from fugu, human and medaka. The protein sequence of fugu Amhr2^D384^ was aligned with human and medaka Amhr2 by ClustalW. Arrow heads indicate intron positions in fugu *Amhr2^D384^*. The kinase domain of fugu Amhr2^D384^ was predicted using SMART (http://smart.embl-heidelberg.de/) and is boxed in black. Amino acid polymorphisms were observed at N/I285 (shown in orange) and D/H384 (boxed in red) between two allelic products of fugu *Amhr2* (*Amhr2^D384^* and *Amhr2^H384^*). Examination of genomic sequences of multiple individuals suggested that while D384 is perfectly correlated with Y chromosome, N285 is not (Table S4B, SNP7864). The position of the amino acid mutation in the medaka (*hotei*) that results in a female phenotype when homozygous is boxed in green [22]. The positions of natural mutations leading to loss-of-function of AMHR2 in human are boxed in cyan [26]. (B) Syntenic blocks around *Amhr2* in fugu, *Tetraodon*, stickleback, medaka and human. Orthologous genes (box) are connected by lines. Orthologs shared by human and fishes are indicated by blue boxes. In medaka, *Amhr2* appears to have been transposed from Chr 5 to Chr 7. Genome sequences in the Ensembl database were used for the synteny analysis (www.ensembl.org; Fugu version 4, TETRAODON 7, stickleback (BroadS1), MEDAKA 1, GRCh37). (C) Neighbour-Joining tree of Amhr2 and its related proteins. Values at the nodes represent bootstrap analysis of 1,000 replicates. The phylogeny confirms the orthologous relationship of fugu Amhr2 to Amhr2 in other vertebrates. There is only one copy of *Amhr2* gene in fugu, *Tetraodon*, stickleback and medaka. *Amhr2* gene is missing in the zebrafish genome database. There are two copies of *Bmpr2* in the zebrafish genome, most likely due to the fish-specific genome duplication. However, only one *Bmpr2* gene is present in fugu and *Tetraodon*. *Acvr2* family has two members each in the human and mouse genomes (*Acvr2A* and *Acvr2B*). Ensembl IDs of sequences used: fugu Amhr2, AB618627; Tetraodon Amhr2, GSTENP00013590001; medaka Amhr2, NM_001104728; stickleback Amhr2, ENSGACT00000008852; mouse Amhr2, NP_653130; human AMHR2, Q16671; fugu Acvr2A_1, QENSTRUT00000010975; fugu Acvrr2A_2, QENSTRUT00000004179; fugu Acvr2A_3, QENSTRUT00000002216; Tetraodon Acvr2A, ENSTNIT00000020534; zebrafish Acvr2A_1, ENSDART00000025989; zebrafish Acvr2A_2, ENSDART00000002428; mouse Acvr2A, ENSMUST00000063886; human ACVR2A, ENST00000404590; fugu Acvr2B, ENSTRUT00000009901; Tetraodon Acvr2B, ENSTNIT00000018138; zebrafish Acvr2B_1, ENSDART00000060866; zebrafish Acvr2B_2, ENSDART00000019975; mouse Acvr2B, ENSMUST00000035093; human ACVR2B, ENST00000352511; fugu Bmpr2, ENSTRUG00000006256; Tetraodon Bmpr2, ENSTNIT00000016485; medaka Bmpr2, ENSORLG00000003034; zebrafish Bmpr2a, NM_001039817; zebrafish Bmpr2b, NM_001039807; mouse Bmpr2 NP_031587; human BMPR2, NP_001195. (D) A homology-based 3D model of the kinase domain of fugu Amhr2. The primary structures of the fugu Amhr2 was deduced from cDNA sequences. The position of H/D384 associated with the phenotypic sex in fugu is shown in red. The position of the amino acid corresponding to the mutation in *hotei* medaka is shown in green [22]. The four amino acids and one segment corresponding to the known positions of natural mutations in human AMHR2 are shown in cyan [26]. To construct a homology-based model of fugu Amhr2, we searched structural templates on the SWISSMODEL [47] servers and found the human BMPR2 amino acid sequence (PDB number, 3g2fA). All structural analyses and graphical representations of molecules were performed using Swiss-Pdb Viewer [48].
(TIF)

Table S1 Genetic position and direction of scaffolds in and around the SD region of fugu.
(XLS)

Table S2 Summary of pedigreed families and the genotype of SNP7271 in them.
(XLS)

Table S3 Screening for variants.
(XLS)

Table S4 Marker information for association test.
(XLS)

Table S5 Primer sequences.
(XLS)
