# Supplementary figures and images for: A Trans-Species Missense SNP in Amhr2 Is Associated with Sex Determination in the Tiger Pufferfish, Takifugu rubripes (Fugu)
Source: PLoS Genet. 2012 Jul 12;8(7):e1002798. doi: 10.1371/journal.pgen.1002798 (PMC3395601; doi:10.1371/journal.pgen.1002798)

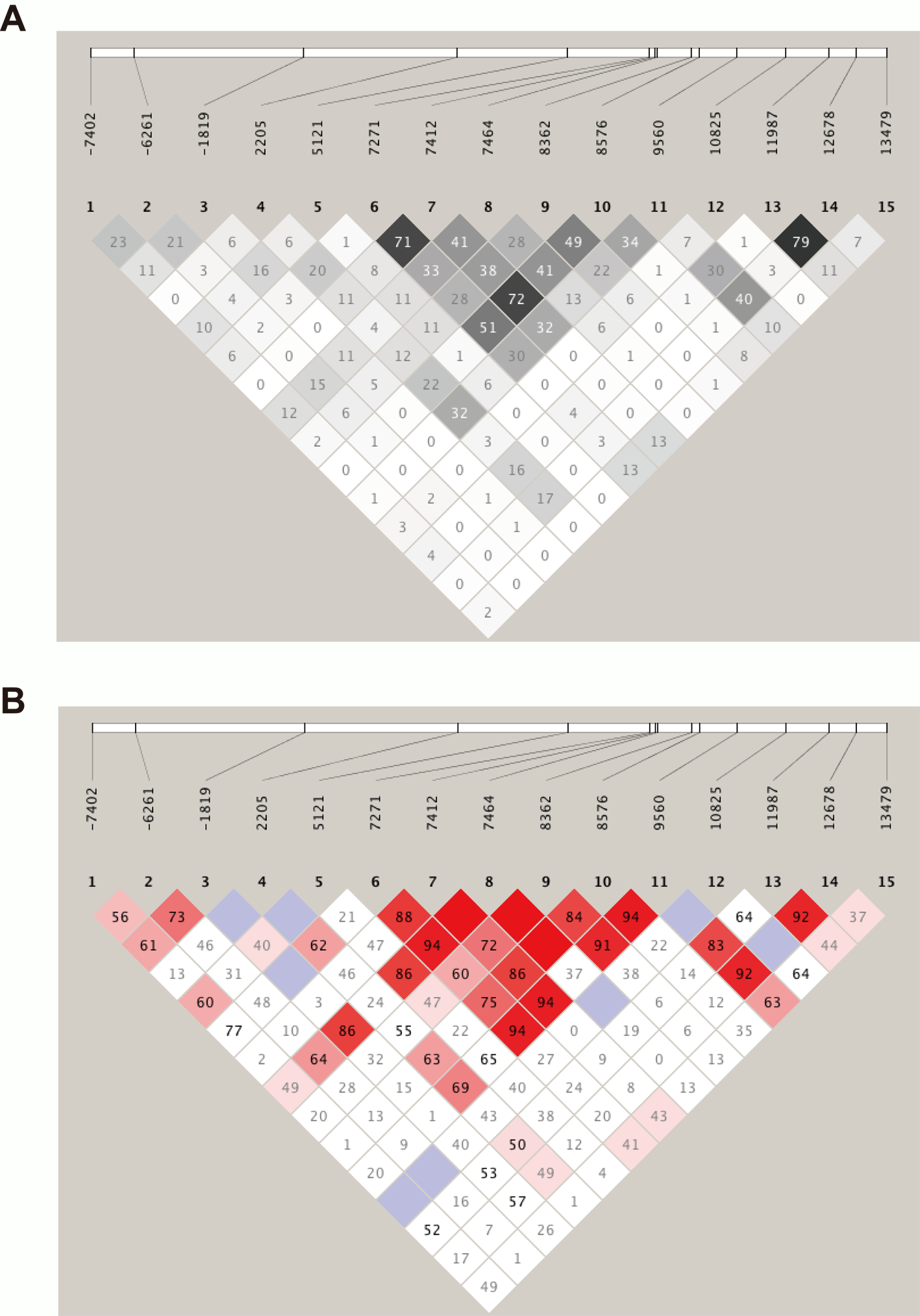

Supplement: Figure S2 — Linkage disequilibrium of the SD region of fugu. r2 and D′ values ×100 estimated for each pairwise comparison of SNPs are shown in plots (A) and (B), respectively. Darker grey in (A) indicates higher r2 (white, r2 = 0; shades of grey, 0<r2<1; black, r2 = 1), while darker red in B indicates higher D′ (white, D′<1 and LOD<2; blue, D′ = 1 and LOD<2; pink, D′<1 and LOD≥2; red, D′ = 1 and LOD≥2). (TIF) [file pgen.1002798.s002.tif]

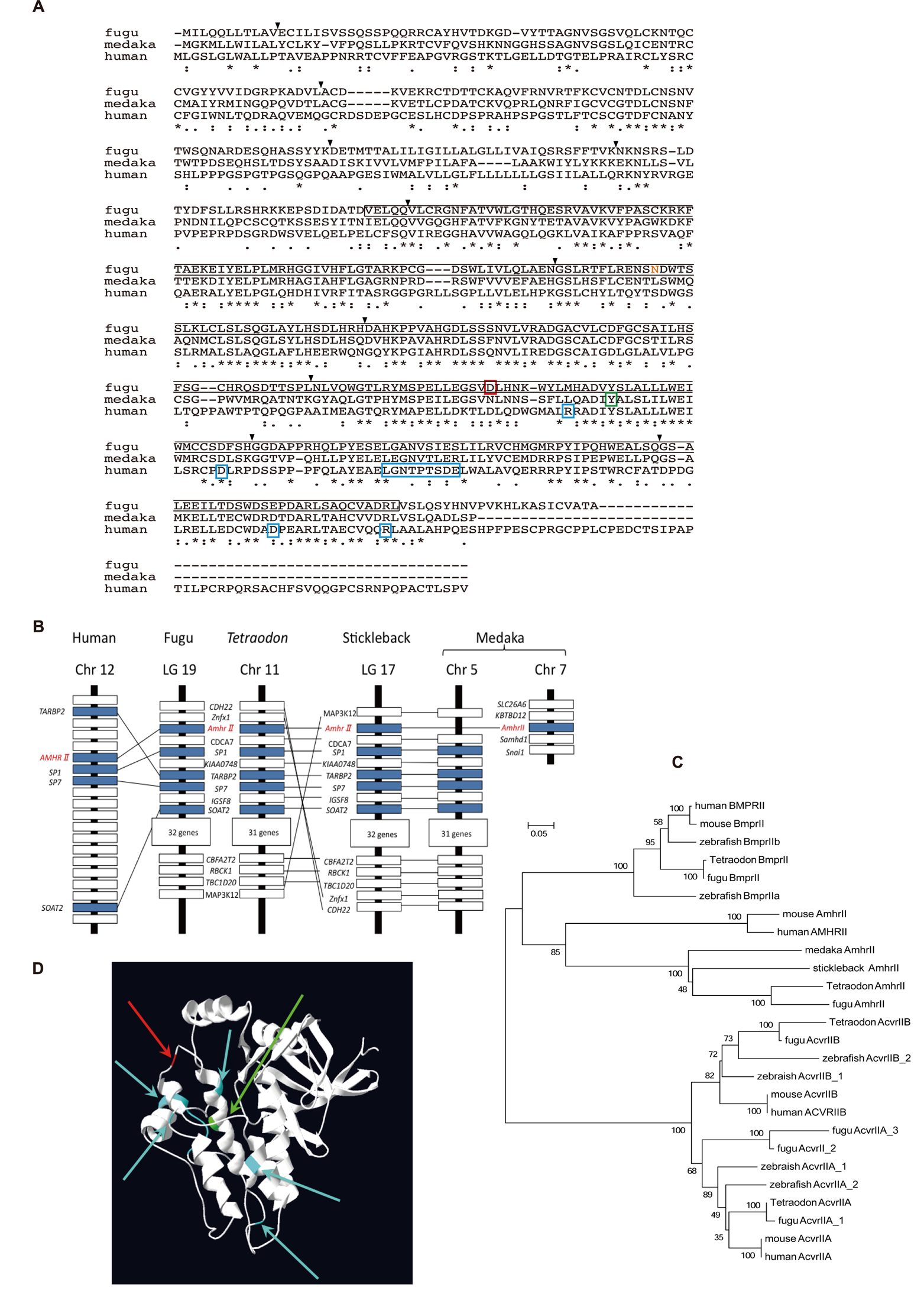

Supplement: Figure S4 — Trans-species comparison of Amhr2. (A) Comparison of Amhr2 from fugu, human and medaka. The protein sequence of fugu Amhr2D384 was aligned with human and medaka Amhr2 by ClustalW. Arrow heads indicate intron positions in fugu Amhr2D384. The kinase domain of fugu Amhr2D384 was predicted using SMART (http://smart.embl-heidelberg.de/) and is boxed in black. Amino acid polymorphisms were observed at N/I285 (shown in orange) and D/H384 (boxed in red) between two allelic products of fugu Amhr2 (Amhr2D384 and Amhr2H384). Examination of genomic sequences of multiple individuals suggested that while D384 is perfectly correlated with Y chromosome, N285 is not (Table S4B, SNP7864). The position of the amino acid mutation in the medaka (hotei) that results in a female phenotype when homozygous is boxed in green [22]. The positions of natural mutations leading to loss-of-function of AMHR2 in human are boxed in cyan [26]. (B) Syntenic blocks around Amhr2 in fugu, Tetraodon, stickleback, medaka and human. Orthologous genes (box) are connected by lines. Orthologs shared by human and fishes are indicated by blue boxes. In medaka, Amhr2 appears to have been transposed from Chr 5 to Chr 7. Genome sequences in the Ensembl database were used for the synteny analysis (www.ensembl.org; Fugu version 4, TETRAODON 7, stickleback (BroadS1), MEDAKA 1, GRCh37). (C) Neighbour-Joining tree of Amhr2 and its related proteins. Values at the nodes represent bootstrap analysis of 1,000 replicates. The phylogeny confirms the orthologous relationship of fugu Amhr2 to Amhr2 in other vertebrates. There is only one copy of Amhr2 gene in fugu, Tetraodon, stickleback and medaka. Amhr2 gene is missing in the zebrafish genome database. There are two copies of Bmpr2 in the zebrafish genome, most likely due to the fish-specific genome duplication. However, only one Bmpr2 gene is present in fugu and Tetraodon. Acvr2 family has two members each in the human and mouse genomes (Acvr2A and Acvr2B). Ensembl IDs of seq [file pgen.1002798.s004.tif]
